# New records of two endemic troglobitic and threatened arachnids (Amblypygi and Opiliones) from limestone caves of Minas Gerais state, southeast Brazil

**DOI:** 10.3897/BDJ.3.e5260

**Published:** 2015-11-10

**Authors:** Bruno Gabriel O do Monte, Jonas Eduardo Gallão, Diego M von Schimonsky, Maria Elina Bichuette

**Affiliations:** ‡ Laboratório de Estudos Subterrâneos, Universidade Federal de São Carlos, São Carlos, São Paulo, Brazil; § Programa de Pós-Graduação em Biologia Comparada-FFCLRP-USP, Ribeirão Preto, São Paulo, Brazil

**Keywords:** *
Charinus
*, *
Iandumoema
*, new distribution, Olhos d’Água cave, Lapa do Cipó cave

## Abstract

**Background:**

The endemic and threatened troglobites (organisms restricted to caves) *Charinus eleonorae* (Amblypygi) and *Iandumoema uai* (Opiliones), both from Olhos d’Água cave, located at Peruaçu Caves National Park (southeast Brazil), have their distribution expanded for a new locality inside of the National Park (Lapa do Cipó cave), extending their distribution at least in 9.5km^2^.

**New information:**

This new data suggest that these arachnids can be in a differentiation process and/or there are several possibilities of dispersion in the karst of Peruaçu. Indeed, a revision of their categorical status at IUCN Red List is necessary. We herein report a new distribution range (Lapa do Cipó cave) of the troglobitic species *I. uai* and *C. eleonorae*, which are, to date, known to occur in the Olhos d’Água cave, located at the Peruaçu Caves National Park (PCNP).

## Introduction

In Brazil there are thirteen species of troglobitic (obligatory cave-dweller) opilionids, belonging to two families: Gonyleptidae Sundevall, 1833 (*Pachylospeleus strinatii* Šilhavý, 1974; *Iandumoema uai* Pinto-da-Rocha, 1996; *Giupponia chagasi* Pérez & Kury, 2002; *Discocyrtus pedrosoi* Kury, 2008; *Eusarcus elinae* Kury, 2008; *Spinopilar moria* Kury & Pérez-González, 2008; *Iandumoema setimapocu*, Hara & Pinto-da-Rocha, 2008; *Iandumoema* sp. n.; *Eusarcus* sp. n. 2; *Eusarcus* sp. n. 3 and two undescribed Pachylinae) and Escadabiidae Kury and Pérez, 2003 (*Spaeleolepts spaeleus* Soares, 1966) (Hara and Pinto-da-Rocha 2008). Out of these eight troglobitic opilionid species, seven Gonyleptidae are from Bahia, Minas Gerais, and São Paulo states and only one Escadabiidae species is from Minas Gerais State (Trajano and Bichuette 2010).

The troglobitic belonging to the genus *Iandumoema* Pinto-da-Rocha, 1996 consists of three species, *Iandumoema uai* Pinto-da-Rocha, 1996, *Iandumoema setimapocu* Hara and Pinto-da-Rocha, 2008 and *Iandumoema* sp. n. All of the species occur in the center-north of Minas Gerais State and each one of them is recorded only from their type-locality. For example, *I. uai* is found at the Olhos d’Água cave (Pinto-da-Rocha 1996).

The genus Charinus Simon, 1982 is the most diverse in Order Amblypygi and the Family Charinidae Quintero, 1986. This genus comprises 17 species in South America and 11 species in Brazil (Harvey 2003, Vasconcelos et al. 2014). Out of these, two are trogobitic species: *C. troglobius* Baptista and Giupponi, 2002 and *C. eleonorae* Baptista and Giupponi, 2003. *C. eleonorae* like *I. uai* is also known only from its type-locality, the Olhos d’Água cave at Itacarambi municipality in the north of Minas Gerais State.

Out of these, two are trogobitic species: *C. troglobius* Baptista and Giupponi, 2002 and *C. eleonorae* Baptista and Giupponi, 2003.

However, endemism is not necessarily a characteristic of such obligatory cave-dweller arachnids in Brazil, since there are records of troglobitic species occurring in two or more caves in Brazil. For example, the opilionid *Discocyrtus pedrosoi* Kury, 2008 from Chapada Diamantina, central region of Bahia State, occurs in seven caves (J.E. Gallão, pers. obs.) and the opilionid *Giupponia chagasi* Pérez and Kury, 2002, as well as the amblypygid *C. troglobius*, both from Serra do Ramalho karstic area, south of Bahia state, occur in two caves (Baptista and Giupponi 2002).

The ecological importance and fragility of the troglobitic species, *I. uai* and *C. eleonorae*, are recognized by the International Union for Conservation of Nature (IUCN). Both these species are included in the IUCN Red List as Critically Endangered (CR), the higher risk category, highlighting their extremely vulnerability (IUCN 2014). However, neither a management plan nor an access control has been implemented for the caves inside a Peruaçu Caves National Park (PCNP), putting those species in risk.

We herein report a new distribution range (Lapa do Cipó cave) of the troglobitic species *I. uai* and *C. eleonorae*, which are, to date, known to occur in the Olhos d’Água cave, located at the Peruaçu Caves National Park (PCNP).

## Materials and methods

### Study area

The Peruaçu Caves National Park (PCNP) located at the Peruaçu Basin of the São Francisco River Basin, is compound by large rocky outcrops with predominance of limestone rocks of Bambuí karst area (Auler et al. 2001). The region is located between the transition of Cerrado (savannah-like vegetation) and Caatinga (semiarid vegetation) phytophysiognomies (Ab'Saber 1977); the dry season in this region occurs between April and November and the average temperature is 24°C (Nimer 1979).

The Olhos d’Água cave is the largest cave of Minas Gerais State, with approximately nine kilometers of horizontal projection (Auler et al. 2001), occurring at the PCNP. The cave is considered a spot of high biodiversity in Brazil, with more than seven troglobitic species (Deharveng and Bedos 2005, Trajano and Bichuette 2010).

**Figure 1.**
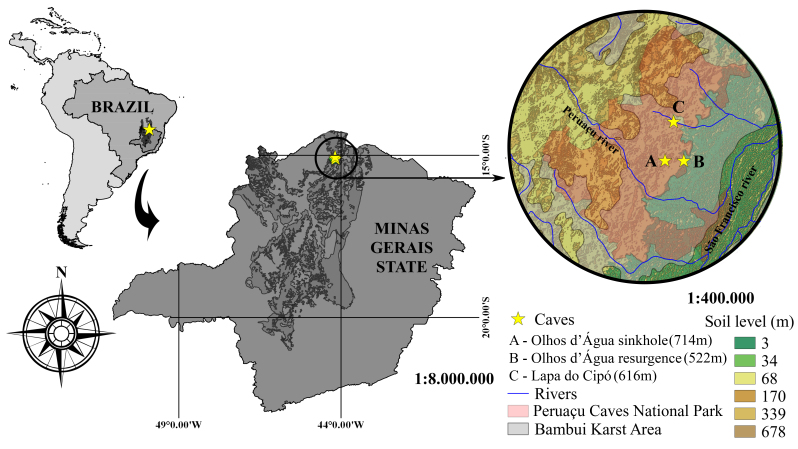
Type-locality (Olhos d’Água cave) and new records (Lapa do Cipó cave) for the troglobitic *Iandumoema uai* and *Charinus eleonorae*. The soil level represents the relative altitudes in the area and the drainages are in the lowest level. Olhos d’Água cave resurgence is the main entrance for this cave. See the two separated drainages for both caves.

**Figure 2.**
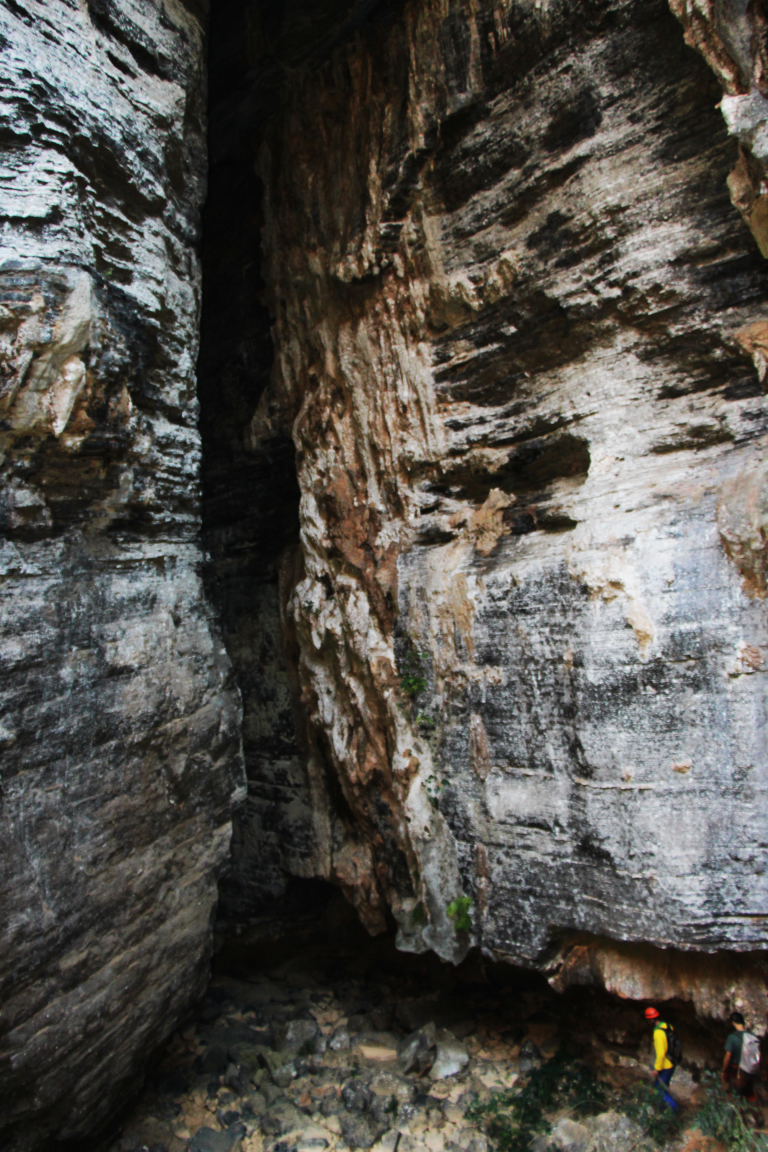
Lapa do Cipó cave entrance; ca. 20 meters high. Both people in the bottom right are for scale.

We conducted fieldtrips to PCNP in June and August of 2014 for sampling caves (Fig. 1). The Lapa do Cipó cave (S 15.05611, W 44.18444) (Fig. 2) is located 6.5 km northwest of the Olhos d’Água cave entrance. Both caves are in different small drainages (part of Peruaçu basin – see Fig. 1) which can be an isolation factor for aquatic fauna, but not necessarily for terrestrial cave invertebrates, however hidrogeological studies are still needed.

To recognize the minimal occurence area of the species, we did a triangulation with the three points of caves (see Fig. 1).

### Collection and identification

We employed the direct qualitative search and hand collecting sampling method targeting walls, under block rocks, organic matter, and unconsolidated substrate (wet and dry). The collected individuals were fixed in 70% ethanol.

Identification and diagnosis of species was conducted following the original description of taxa (Pinto-da-Rocha 1996, Baptista and Giupponi 2003). We compared individuals from Lapa do Cipó cave and those from the type-locality, Olhos d’Água cave. Additional reference material, deposited at Laboratório de Estudos Subterrâneos (LES) collection from Universidade Federal de São Carlos (UFSCar), São Carlos municipality from São Paulo state, were also used for the comparison.

In addition, for body measurements, we used the classical morphometric data specific for each group to confirm the identification. The measurements for opilionids and amblypygids were conducted as described by Acosta et al. (2007) and Quintero-Jr. (1981), respectively. We used a digital caliper with 0.01 mm accuracy for the measurements. In total, we measured 13 specimens of *I. uai* (seven from Olhos d’Água cave and six from Lapa do Cipó cave) and 10 specimens of *C. eleonorae* (seven from Olhos d’Água cave and three from Lapa do Cipó cave).

Images were taken using a Leica DFC 295 camera attached to a Leica M205C stereomicroscope with a PlanApo (1.0) objective. The figures were produced using multiple frames of LAS software (Leica Application Suite v3.7).

### Taxonomy

The genus *Iandumoema* is characterized by a single erect spine on eye mound, areas of dorsal scutum unarmed, and presence of mesal-subapical setae on the pedipalpal femur (Fig. 3a, b, c). The two species of this genus, *I. setimapocu* and *I. uai*, differ in terms of the curvature on femur IV as observed on *I. uai* and the direction of dorso-apical apophyses on male coxae IV; the latter is directed laterally backwards, away from the body in *I. uai*, while it is closer in *I. setimapocu*. In addition, in *I. uai* males, the tibial pedipalpal setae features IiIi conformation (Fig. 3a) (Pinto-da-Rocha 1996, Hara and Pinto-da-Rocha 2008).

The genus *Charinus* is characterized by the following characteristics: pedipalpal basitarsus with two long dorsal spines and one ventral; pedipalpal tibia expanded dorsally, with a spine and a setiferous tubercle distally in relation to its longest dorsal spine; trochanter with a well-developed ventral protuberance, with setiferous tubercles anteriorly projected (Armas and Pérez-González 2001). *Charinus troglobius* is characterized by an anterior depression on the carapace, in place of the absent median eye tubercle (Baptista and Giupponi 2002), whereas *C. eleonorae* (Fig. 4a, b, c) is characterized by an indistinct median eye tubercle (Fig. 4a, b, c) usually with only two very small eye spots, or without eye spots in rare cases (Baptista and Giupponi 2003).

## Taxon treatments

### Charinus eleonorae

Baptista & Giupponi 2003

#### Materials

- **Type status:** Other material. **Occurrence:** occurrenceDetails: Olhos d'Água cave; individualCount: 5; **Taxon:** taxonID: Charinus eleonorae; acceptedNameUsageID: Charinus eleonorae; **Location:** locationID: Laboratório de Estudos Subterrâneos / Universidade Federal de São Carlos; higherGeographyID: São Carlos, São Paulo State; higherGeography: Brazil; continent: South America; verbatimCoordinates: 15 06 49.0S 44 10 10.0W; geodeticDatum: WGS84; **Identification:** identificationID: LES 0400; LES03217;; identifiedBy: Bruno Gabriel O. do Monte; identificationReferences: Baptista & Giupponi 2003; **Geological context:** geologicalContextID: Bambuí geomorphological unit, limestone from Peruaçu karst area, Medium São Francisco basin, southeast Brazil; **Event:** eventID: July 26, 2010
- **Type status:** Other material. **Occurrence:** occurrenceDetails: Olhos d'Água cave; individualCount: 2; **Taxon:** taxonID: Charinus eleonorae; acceptedNameUsageID: Charinus eleonorae; **Location:** locationID: Laboratório de Estudos Subterrâneos / Universidade Federal de São Carlos; higherGeographyID: São Carlos, São Paulo State; higherGeography: Brazil; continent: South America; verbatimCoordinates: 15 06 49.0S 44 10 10.0W; verbatimSRS: WGS84; **Identification:** identificationID: LES03232; LES05873;; identifiedBy: Bruno Gabriel O. do Monte; identificationReferences: Baptista & Giupponi 2003; **Geological context:** geologicalContextID: Bambuí geomorphological unit, limestone from Peruaçu karst area, Medium São Francisco basin, southeast Brazil; **Event:** eventID: July 23-24, 2012
- **Type status:** Other material. **Occurrence:** occurrenceDetails: Lapa do Cipó cave; individualCount: 3; **Taxon:** taxonID: Charinus eleonorae; acceptedNameUsageID: Charinus eleonorae; **Location:** locationID: Laboratório de Estudos Subterrâneos / Universidade Federal de São Carlos; higherGeographyID: São Carlos, São Paulo State; higherGeography: Brazil; continent: South America; verbatimCoordinates: 15 03 22.0S 44 11 04.0W; verbatimSRS: WGS84; **Identification:** identificationID: LES05868; identifiedBy: Bruno Gabriel O. do Monte; identificationReferences: Baptista & Giupponi 2003; **Geological context:** geologicalContextID: Bambuí geomorphological unit, limestone from Peruaçu karst area, Medium São Francisco basin, southeast Brazil; **Event:** eventID: June 05, 2014

#### Conservation

According to 2014 IUCN revision, this species is CR (Critically Endangered) category.

#### Taxon discussion

Expansion of occurrence of troglobitic species previously known for only a single cave.

### Iandumoema uai

Pinto-da-Rocha 1996

#### Materials

- **Type status:** Other material. **Occurrence:** occurrenceDetails: Olhos d'Água cave; individualCount: 7; **Taxon:** taxonID: Iandumoema uai; acceptedNameUsageID: Iandumoema uai; **Location:** locationID: Laboratório de Estudos Subterrâneos / Universidade Federal de São Carlos; higherGeographyID: São Carlos, São Paulo State; higherGeography: Brazil; continent: South America; verbatimCoordinates: 15 06 49.0S 44 10 10.0W; verbatimSRS: WGS84; **Identification:** identificationID: LES03214, LES03233; identifiedBy: Bruno Gabriel O. do Monte; **Geological context:** geologicalContextID: Bambuí geomorphological unit, limestone from Peruaçu karst area, Medium São Francisco basin, southeast Brazil; **Event:** eventID: July 26, 2010
- **Type status:** Other material. **Occurrence:** occurrenceDetails: Lapa do Cipó cave; individualCount: 2; sex: MALE; **Taxon:** taxonID: Iandumoema uai; acceptedNameUsageID: Iandumoema uai; **Location:** locationID: Laboratório de Estudos Subterrâneos / Universidade Federal de São Carlos; higherGeographyID: São Carlos, São Paulo State; higherGeography: Brazil; continent: South America; verbatimCoordinates: 15 03 22.0S 44 11 04.0W; verbatimSRS: WGS84; **Identification:** identificationID: LES05869, LES05870; identifiedBy: Bruno Gabriel O. do Monte; **Geological context:** geologicalContextID: Bambuí geomorphological unit, limestone from Peruaçu karst area, Medium São Francisco basin, southeast Brazil; **Event:** eventID: June 05, 2014
- **Type status:** Other material. **Occurrence:** occurrenceDetails: Lapa do Cipó cave; individualCount: 4; sex: MALE; **Taxon:** taxonID: Iandumoema uai; acceptedNameUsageID: Iandumoema uai; **Location:** locationID: Laboratório de Estudos Subterrâneos / Universidade Federal de São Carlos; higherGeographyID: São Carlos, São Paulo State; higherGeography: Brazil; continent: South America; verbatimCoordinates: 15 03 22.0S 44 11 04.0W; verbatimSRS: WGS84; **Identification:** identificationID: LES05871, LES05872; identifiedBy: Bruno Gabriel O. do Monte; **Geological context:** geologicalContextID: Bambuí geomorphological unit, limestone from Peruaçu karst area, Medium São Francisco basin, southeast Brazil; **Event:** eventID: August 26, 2014

#### Conservation

According to 2014 IUCN revision, this species is CR (Critically Endangered) category.

#### Taxon discussion

Expansion of occurrence of troglobitic species previously known for only a single cave.

## Analysis

Individuals of *I. uai* and *C. eleonorae*, recorded from Lapa do Cipó cave, are the first reported occurrence of these species from any other cave beyond their type-locality, Olhos d’Água cave. We observed that all individuals from both the species present the diagnostic characters for *I. uai* and *C. eleonorae*, with no sharp differences in characters among the specimens from both localities. However, all the three *C. eleonorae* specimens from Lapa do Cipó cave had only eyespots with the median eyes absent, which are reduced in the specimens from Olhos d’Água cave. Morphometric data of both the species from Lapa do Cipó cave are in agreement with the original description with subtle differences (Tables 1, 2).

## Discussion

The Olhos d’Água cave is the largest cave in the Minas Gerais State with a significant number of troglobitic species and restricted range distribution (Deharveng and Bedos 2005, Trajano and Bichuette 2010). Nevertheless, there is no information on other entrances or accesses except the known resurgence. Therefore, we considered that the hypogean habitats are isolated. However, the Olhos d’Água cave upstream entrance (sinkhole), known as Água d’Olhos (Piló 1989) may represent an alternative passageway to the cave fauna. To date, there are no geological studies that indicate a possible communication with the other caves in the region.

The occurrence of *I. uai* and *C. eleonorae* in Lapa do Cipó cave (distant 6.5 km northwest from Olhos d'Água cave) either indicates the existence of a complex system of subterranean microspaces (such as cracks and fissures), interconnecting both caves in the karst of Peruaçu; or the past existence of a whole unique system, reaching these two caves. In the first case, the dispersion can occur through the voids connected through cracks and fissures, typical of the MSS habitat (mesovoid shallow substratum, sensu Juberthie 2000). In the latter case, as defined by Ford and Williams 2007, which outlined that cave systems are integrated habitats linking output and input points of dissolution, which possibilities the flow of clastic sediments and, particularly, the fauna. These two hypothesis are not mutually exclusive.

In terms of median eyes, we observed that the *C. eleonorae* populations from Lapa do Cipó cave showed a higher frequency of individuals without them comparing to the observed for Olhos d’Água cave population. The absence of median eyes is considered a rare condition by (Baptista and Giupponi 2003). Our data (frequent absence of median eyes) strongly suggests that this variation must be more common than the authors considered and the description and proposed key differenting *C. eleonorae* and *C. troglobius* must be reviewed. However, such variability has not been recorded for the highly troglomorphic species, *C. troglobius*, from Serra do Ramalho (Bahia), and therefore, in this case, the absence of median eyes is a robust diagnostic feature (Baptista and Giupponi 2003). Furthermore, the occurrence of such variability in *C. eleonorae* is a indicative of or relaxed pressure or even neutralism processes, testable hypothesis through population genetic studies.

For conservation purposes, the data presented herein, such as the occurrence area of 9.5km^2 ^made by triangulation of A, B and C points (Fig. 1), suggests a revision of the category status of these two species in the IUCN list (from critically endangered - CR to Endangered - EN). Finally, an implementation of the management plan for caves in the Peruaçu Caves National Park (PCNP) is urgent, as well as the regulation of cave tourism in the region.

## Supplementary Material

XML Treatment for Charinus eleonorae

XML Treatment for Iandumoema uai

## Figures and Tables

**Figure 3a.**
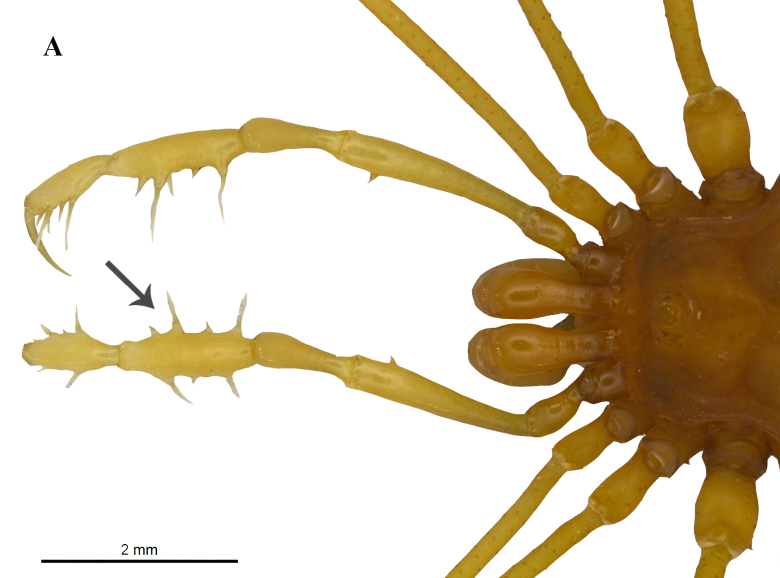
Dorsal view of pedipalpal tibia-tarsus ectal and mesal setae IiIi

**Figure 3b.**
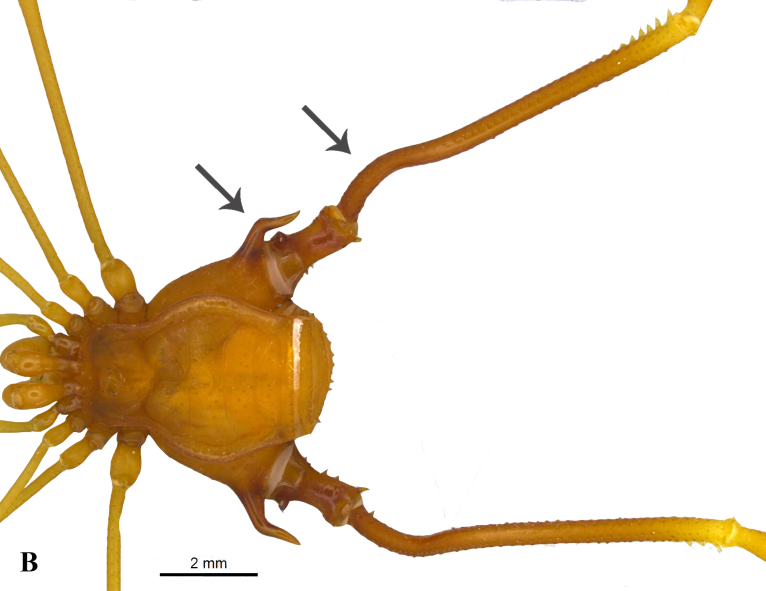
Dorsal view of habitus, showing coxa IV tuberculate laterally, with robust apical external apophysis and curved at 1/3 distal (on male) and femurIV curved laterally and dorsally at 1/3 from base (on male)

**Figure 3c.**
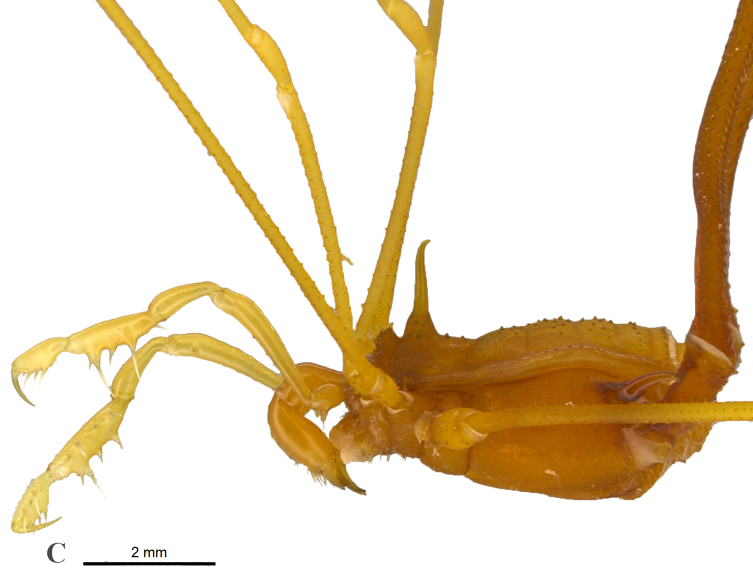
Lateral view of habitus, showing eye mound with erect high spine, with acuminate apex pointing slightly backwards.

**Figure 4a.**
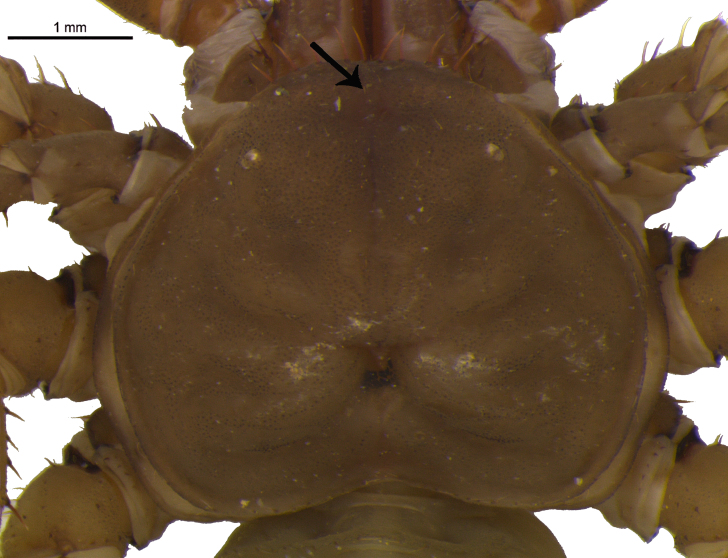
Carapace, dorsal view, showing mid-portion of carapace without median eyes

**Figure 4b.**
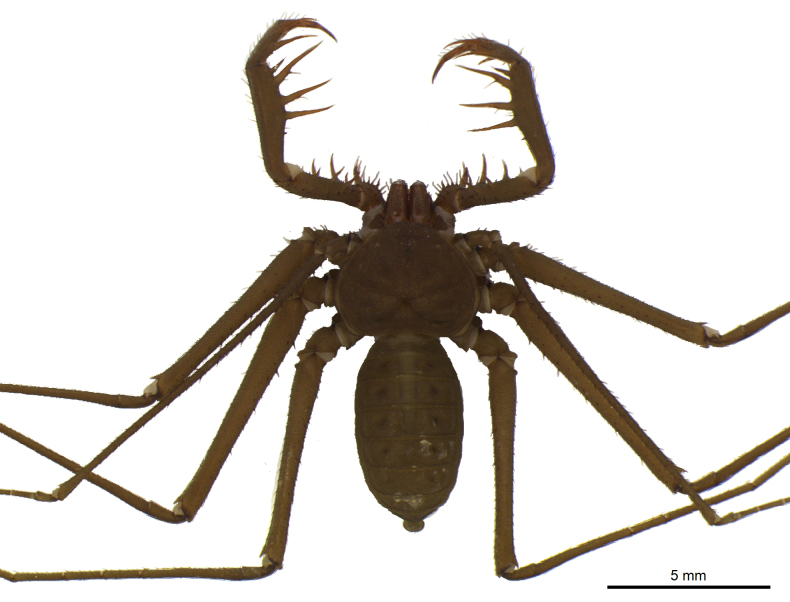
Habitus, dorsal view

**Figure 4c.**
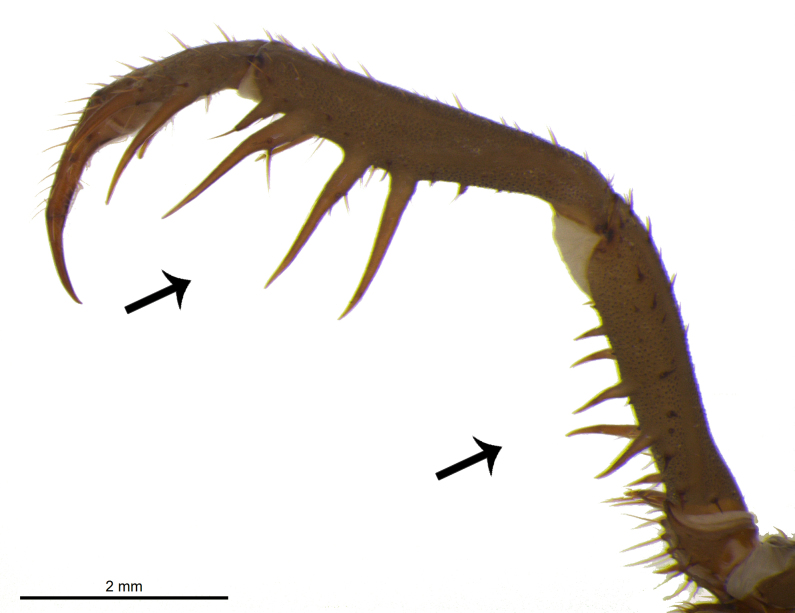
Lateral view of right pedipalp showing spines.

**Table 1.** Mean measurements values ​​and standard error for the opilionid *Iandumoema uai* from Olhos d’Água and Lapa do Cipó caves. The fifth column brings the data of the original description – type-series (Pinto-da-Rocha 1996).

		Olhos d’Água n = 7	Lapa do Cipó n = 6	Type-series n = 9
Pedipalpus	Trochanter	0.48(0.01)	0.52(0.02)	0.54
	Femur	2.03(0.05)	1.92(0.08)	1.96
	Patella	1.01(0.02)	1(0.04)	1.10
	Tibia	1.46(0.04)	1.33(0.04)	1.40
	Tarsus	1.01(0.03)	1(0.03)	1.10
	Total	6(0.11)	5.78(0.18)	6.10
Leg I	Trochanter	0.54(0.02)	0.528(0.03)	0.46
	Femur	4.57(0.06)	4.09(0.18)	4.40
	Patella	1.08(0.02)	0.989(0.03)	1.12
	Tibia	3.51(0.07)	3.17(0.08)	3.37
	Metatarsus	5.83(0.03)	5.26(0.2)	5.69
	Tarsus	2.48(0.24)	3.72(1.1)	2.75
	Total	18.03(0.26)	17.76(1.1)	17.79
Leg II	Trochanter	0.60(0.02)	0.56(0.03)	0.60
	Femur	9.24(0.07)	8.12(0.3)	8.64
	Patella	1.61(0.01)	1.27(0.07)	1.44
	Tibia	8.05(0.02)	7.26(0.2)	7.37
	Metatarsus	9.44(0.08)	8.72(0.22)	8.88
	Tarsus	10.16(0.18)	9.75(0.14)	9.84
	Total	37.31(1.3)	32.63(3.56)	36.77
Leg III	Trochanter	0.61(0.03)	0.58(0.03)	0.62
	Femur	6.21(0.06)	5.6(0.18)	5.70
	Patella	1.22(0.02)	1.13(0.04)	1.28
	Tibia	3.88(0.05)	3.56(0.11)	3.56
	Metatarsus	6.55(0.08)	6.03(0.21)	6.31
	Tarsus	3.10(0.05)	2.98(0.61)	2.87
	Total	21.60(0.17)	18.24(1.57)	20.34
Leg IV	Trochanter	1.05(0.03)	0.99(0.16)	1.15
	Femur	8.17(0.06)	7.6(0.26)	8.10
	Patella	1.42(0.07)	1.42(0.09)	1.69
	Tibia	5.94(0.07)	5.37(0.21)	5.56
	Metatarsus	8.85(0.19)	8.25(0.28)	8.56
	Tarsus	3.84(0.02)	3.75(0.09)	3.59
	Total	29.29(0.4)	25.40(2.03)	28.65
Dorsal scute length		3.86(0.07)	3.7(0.15)	4.04
Prosoma length		4.40(0.09)	3.55(0.08)	1.52
Prosoma width		3.77(0.29)	3.72(0.24)	1.88
Opisthosoma width		2.44(0.04)	2.13(0.2)	3.16

**Table 2.** Mean measurements values ​​and standard error for the amblypigid *Charinus eleonorae* from Olhos d’Água and Lapa do Cipó caves. The fourth column brings the data of the original description – type-series (Baptista and Giupponi 2003).

	Olhos d’ Água n = 7	Lapa do Cipó n = 3	Type-series n = 17
Femur	2.89 (0.36)	3.09(0.01)	4.2(3.4-5.6)
Tibia	3.47(0.43)	3.86(0.22)	4.0(3.2-5.4)
Basitarsus	1.47(0.17)	1.76(0.11)	1.8 (1.6-2.2)
Distitarsus	1.15(0.07)	1.35(0.06)	1.2(1.1-1.4)
Tarsal claw	0.73(0.03)	0.85(0.01)	0.9(0.8-1.1)
Total	9.74(1.02)	10.93(0.36)	
Cephalotorax length	2.97(0.22)	3.23(0.32)	3.5(3.1-4.0)
Cephalotorax width	3.15(0.25)	3.52(0.19)	4.3(3.9-5.1)
Abdomen length	4.60(0.43)	6.17(0.24)	5.0(4.2-5.6)
Body length	7.41(0.58)	9.12(0.37)	7.8(7.0-9.1)

## References

[B1466034] Ab'Saber Aziz Nacib (1977). Os domínios morfoclimáticos na América do Sul. Geomorfologia.

[B1466269] Acosta L. E., Pérez-Gonzalez A., Tourinho A. L., Machado G., Pinto-da-Rocha R., Giribet G. (2007). Methods and Techniques of Study: Methods for taxonomic study. Harvestmen, the biology of Opiliones.

[B1956696] Armas Luis Fernando, Pérez-González Abel (2001). Los amblipígidos de República Dominicana (Arachnida: Amblypygi). Revista Ibérica de Aracnología.

[B1466044] Auler Augusto, Rubbioli Ezio, Brandi Roberto (2001). As grandes cavernas do Brasil.

[B1466053] Baptista Renner Luis Cesar, Giupponi Alessandro Ponce Leão (2002). A new troglomorphic Charinus from Brazil. Revista Ibérica de Aracnologia.

[B1466064] Baptista R. L. C., Giupponi A. P. L (2003). A new troglomorphic Charinus from Minas Gerais State, Brazil (Arachnida: Amblypygi: Charinidae). Revista Ibérica de Aracnologia.

[B1466074] Deharveng Louis, Bedos Anne (2005). Diversity Patterns in the Tropics. Encyclopedia of Caves.

[B1956759] Ford Derek, Williams Paul (2007). Karst hydrogeology and geomorphology.

[B1466088] Hara M. R., Pinto-da-Rocha R. (2008). A new species of Brazilian troglobitic harvestman of the genus Iandumoema (Opiliones: Gonyleptidae). Zootaxa.

[B1466164] Harvey M. S. (2003). Catalogue of the smaller arachnid orders of the world, Amblypygi, Uropygi, Schizomida, Palpigradi, Ricinulei and Solifugae.

[B1466173] IUCN Guidelines for Using the IUCN Red List Categories and Criteria. http://jr.iucnredlist.org/documents/RedListGuidelines.pdf.

[B1956722] Juberthie C., WILKENS H., CULVER D. C., HUMPHREYS W. F. (2000). The diversity of the karstic and pseudo karstic hypogean habitats in the world.. Ecosystems of the World: Subterranean Ecosystems.

[B1466192] Nimer E. (1979). Climatologia do Brasil.

[B1466211] Piló L. B. (1989). A morfologia cárstica do baixo curso do rio Peruaçu, Januária-Itacarambi, MG (Graduate Monography)..

[B1466220] Pinto-da-Rocha Ricardo (1996). Iandumoema uai, a new genus and species of troglobitic harvestman from Brazil (Arachnida, Opiliones, Gonyleptidae). Revista Brasileira de Zoologia.

[B1466230] Quintero-Jr. D. (1981). The amblypygid *Phrynus* in the Americas (Amblypygi, Phrynidae). Journal of Arachnology.

[B1466249] Trajano E., Bichuette M. E. (2010). Diversity of Brazilian subterranean invertebrates with a list of troglomorphic taxa. Subterranean Biology.

[B1466259] Vasconcelos A. C.O., Giupponi A. P. L., Ferreira R. L. (2014). A new species of Charinus from Minas Gerais State, Brazil, with comments on its sexual dimorphism (Arachnida: Amblypygi: Charinidae). Journal of Arachnology.

